# Physical Capacity and Activity in Patients With Idiopathic Normal Pressure Hydrocephalus

**DOI:** 10.3389/fneur.2022.845976

**Published:** 2022-03-28

**Authors:** Johanna Rydja, Lena Kollén, Martin Ulander, Mats Tullberg, Fredrik Lundin

**Affiliations:** ^1^Department of Activity and Health and Department of Biomedical and Clinical Sciences, Linköping University, Linköping, Sweden; ^2^Department of Clinical Neuroscience, Sahlgrenska Academy, University of Gothenburg, Gothenburg, Sweden; ^3^Department of Clinical Neurophysiology and Department of Biomedical and Clinical Sciences, Linköping University, Linköping, Sweden; ^4^Department of Neurology and Department of Biomedical and Clinical Sciences, Linköping University, Linköping, Sweden

**Keywords:** idiopathic normal pressure hydrocephalus, physical activity, exercise, actigraphy, gait, sleep

## Abstract

**Introduction:**

Most patients with idiopathic normal pressure hydrocephalus (iNPH) improve gait after surgery. However, knowledge on physical capacity and activity after shunt surgery is limited. One of the aims of this study was to evaluate the effect of shunt surgery in patients with iNPH on short-distance walking, functional exercise capacity, functional strength, and variables of activity and sleep, 3 and 6 months postoperatively. Another aim was to evaluate the effect of a physical exercise program. Additionally, we studied how changes in short-distance walking were correlated with functional exercise capacity and voluntary walking.

**Methods:**

In total, 127 patients were consecutively included and randomized to the exercise group (*n* = 62) or the control group (*n* = 65). Participants in the exercise group underwent the supervision of a 12-week exercise program. All patients were assessed before surgery, at 3 and 6 months postoperatively with the 10-m walk test (10MWT), the 6-min walk test (6MWT), 30-s chair stand test (30sCST), and with the actigraphic recordings of activity variables measured for a total of 24 h/day for at least 3 days.

**Results:**

All patients improved at 3 months postoperatively in the 10MWT (*p* < 0.001), 6MWT (*p* < 0.001), and 30sCST (*p* < 0.001). These results were maintained after 6 months. Actigraphic recordings for voluntary walking (steps per minute) were improved and nighttime sleep (%) increased after 6 months (*p* = 0.01, *p* = 0.04). There were no significant differences between the exercise group and the control group, except for the postoperative change in the proportion of daytime sleep after 3 months, which was slightly more reduced compared to baseline in the exercise group (*p* = 0.04). Changes after 3 months in the 10MWT and 6MWT were moderately correlated (ρ= −0.49, *p* = 0.01) whereas the correlation between the 10MWT and voluntary walking was weak (ρ = −0.34, *p* = 0.01).

**Conclusion:**

Shunt surgery improved short-distance walking, functional exercise capacity, functional strength, and voluntary walking. An exercise program did not affect these outcomes. Short-distance walking was weakly correlated with voluntary walking, indicating improved physical capacity does not directly translate to increased physical activity. Further research should address how interventions should be tailored to promote physical activity after shunt surgery.

**Trial Registration:**

clinicaltrials.gov, Id: NCT02659111.

## Introduction

The main symptoms of idiopathic normal pressure hydrocephalus (iNPH), caused by disturbance in the cerebrospinal fluid dynamics, are impaired gait and balance, along with cognitive impairment and/or incontinence (1, 2). The available treatment is a shunt insertion, and gait symptoms measured with a timed walking test will improve in approximately 80% of shunted patients (3–5).

We have limited knowledge about the overall activity level of patients before and after shunt surgery. After surgery, patients with iNPH have reported worse health-related quality of life in terms of mobility and daily activities than sex- and age-matched controls (6). A previous small study from our center used a body-worn triaxial accelerometer to measure the physical activity of patients with iNPH in daily life. In that study, patients were less active and took fewer steps with a lower total energy expenditure (TEE) than healthy individuals both before and after shunt surgery; however, no increase in physical activity was observed after surgery (7). Patients with iNPH improve gait as measured by timed short-distance walking tests (3–5), but it is unclear if they improve in terms of overall physical activity in daily life. The impact of shunt surgery on patients' physical capacity (strength and exercise capacity) and activity in daily life has not been thoroughly studied. The remaining questions to be answered are if these variables improve after surgery, and if short-distance walking correlates with exercise capacity and voluntary walking in daily life.

In a randomized clinical trial reported elsewhere (the iNPhys study), we evaluated the immediate and long-term effect of a 12-week high-intensity functional exercise program (HIFE) (8) compared to a control group of patients with iNPH receiving no intervention. The exercise group showed long-term effects on balance and achieved their stated goals to a higher extent than the controls; however, no effect was found on the primary outcome, the total iNPH scale score (9). In this study, we investigated secondary outcomes from the iNPhys study.

One of the aims of this study was to evaluate short-distance walking, functional exercise capacity, functional strength, and activity and sleep variables before, 3 and 6 months after shunt surgery in patients with iNPH. Another aim was to evaluate the effect of a physical exercise program on the same variables. Additionally, we studied how the changes from baseline in short-distance walking were correlated to the changes in functional exercise capacity and voluntary walking, respectively.

## Materials and Methods

### Study Design and Inclusion of Participants

The iNPhys study (9) was a double-center trial conducted at Linköping University hospital in Linköping and Sahlgrenska University hospital in Gothenburg, Sweden. In total, 127 patients diagnosed with iNPH according to international guidelines (1), and planned for shunt surgery were consecutively enrolled between January 2016 and June 2018. Exclusion criteria were Mini-Mental State Examination (MMSE) <16, inability to walk with or without walking aids for >10 m, or suffering from other diseases making intensive exercises impossible. After the decision on surgery, eligible patients were included and randomized to either no intervention or a supervised highly intensive functional exercise intervention, the HIFE^TM^-program (8), two times weekly for 12 weeks. Assessments were performed in the evaluation process before surgery, 3 and 6 months postoperation. All patients had a ventriculo-peritoneal shunt. More details are described in our previous publication (9).

The trial was approved by the medical ethical committee of Linköping, 2015/250-31, and the study protocol was registered in advance at clinicaltrials.gov, Id: NCT02659111. The study received ethical board approval and conforms with the World Medical Association Declaration of Helsinki, and all participants received oral and written information and gave written consent prior to the start of the study.

### Outcome Measures

#### Short-Distance Walking

Short-distance walking was measured with the 10-m walk test (10MWT), in which patients walked 10 m between the two markings at their self-selected speed (10). The mean value in seconds from the two repeated tests was calculated. Patients were able to use their walking aids if needed.

#### Functional Exercise Capacity

Functional exercise capacity was measured with the 6-min walk test (6MWT) (11). To perform the test, patients were asked to walk as far as possible at a self-selected speed along a marked 30-m distance between the two cones for a period of 6 min. If necessary, walking aids were allowed and, if needed, patients could stop and start the test again during the 6-min period. The assessor walked with patients and gave standardized information every minute. The total distance in meters walked for 6 min was noted.

#### Functional Lower Limb Strength

Functional lower limb strength was measured with the 30-s chair stand test (30sCST) (12). The test started with the patient seated in a chair without armrests. On a signal from the assessor, the patient stood upright and immediately sat down again until the back touched the backrest of the chair. This procedure was repeated as many times as possible in 30 s. The assessor stood close to the patient to avoid problems such as falls, but did not assist the patient in the test. The total number of full standings was noted.

#### Actigraphic Recordings

The overall daily activity was measured with an actigraphic recording using a SenseWear actigraph (BodyMedia, Inc., Pittsburg, PA, USA) worn on the dominant upper arm for 7 days. Patients were told to use the armband during the day and at night, and the recordings were divided into daytime, from 8.00 a.m. to 7.59 p.m., and nighttime from 8.00 p.m. to 7.59 a.m. The variables used in the analyses were the number of steps per minute reflecting voluntary walking, TEE, metabolic equivalent of task (MET), and proportions of time patients were asleep during daytime and nighttime. TEE is the total human energy cost, including the basal metabolic rate, the energy cost of digestion, and the energy cost of physical activity (13). MET is defined as the ratio between the energy expended for a certain activity and the energy used when sitting quietly. One MET is the energy expended sitting quietly and in a two-MET activity the person uses two times as much energy (14). To be included in the analysis, monitoring from at least 3 full days was required.

### Statistical Analysis

The set of subjects included in the analyses were participants with at least one follow-up session after surgery. The normal distribution was tested with the Shapiro–Wilk test, and within-group differences were calculated with the related samples Friedmans two-way analysis of variance (ANOVA) by ranks. Significance values in within-group tests were adjusted by the Bonferroni correction for multiple tests. Differences between the exercise group and the control group were calculated with the Mann–Whitney *U* test for independent samples. Between-group differences in the baseline characteristics were calculated with the chi-squared test for categorical variables or the independent sample *t*-test for continuous variables. The correlation analyses were conducted using Spearman's ρ test or Pearson's correlation. Statistical analyses were performed with IBM SPSS Statistics version 27.0 (IBM Corp., Armonk, NY, USA). A two-tailed *p-*value of ≤ 0.05 was considered as statistically significant.

## Results

### Baseline Characteristics and Dropouts

**Figure 1:**
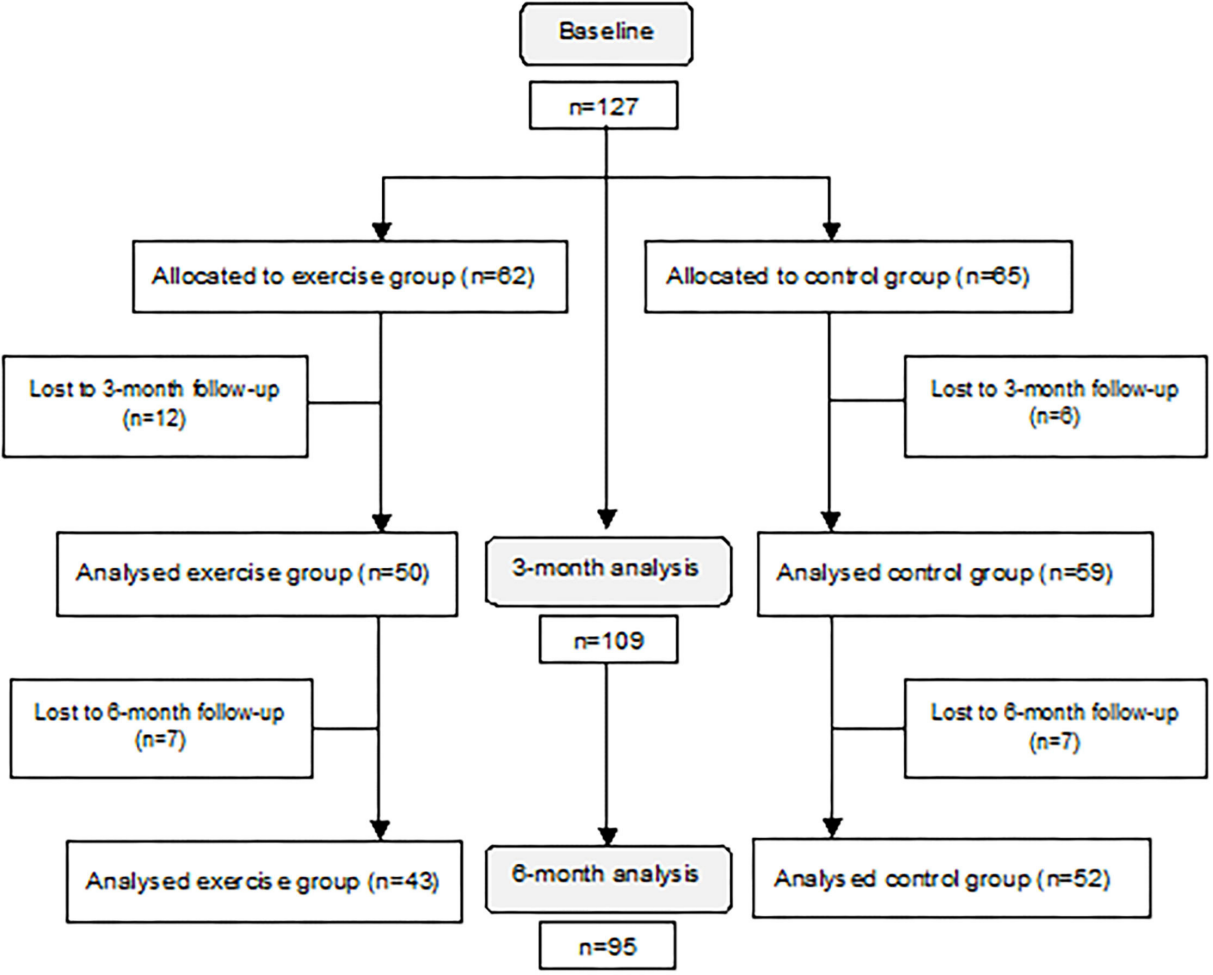
A flowchart of the inclusion and follow-up process, including dropouts.

In total, 127 patients were included in the study. Of these, 109 patients with at least one postoperative efficacy assessment were included in the data analysis (50 patients in the exercise group and 59 patients in the control group). At a follow-up after 6 months, data from 95 patients were available for the analyses (Figure 1). There were no significant differences in baseline characteristics between dropouts and participants included in the data analysis. Among included participants, 59.6% were male, the mean age was 73.7 ± 6.6 years, and the average MMSE result was 26.0 ± 3.0 points. The prevalence figures for comorbidities were: diabetes 31.2%, hypertension 65.1%, cardiovascular diseases 17.4%, stroke 8.3%, and atrial fibrillation 10.1%, and 11.3% of the participants were smokers (Table 1).

**Table 1:** Characteristics of included participants and dropouts at baseline.

	**Participants**	**Dropouts**	***p*-value**
	***n =* 109**	***n =* 18**	
Age (years)	73.7 ± 6.6	73.9 ± 7.4	0.87
Sex (male/female %)	59.6/40.4	50.0/50.0	0.44
BMI	26.6 ± 4.1	27.4 ± 3.8	0.94
MMSE (0–30)	26.0 ± 3.0	25.0 ± 3.0	0.11
Smoking (%)	11.3 (*n =* 106)	11.1	0.98
Diabetes (%)	31.2	22.2	0.44
Hypertension (%)	65.1	77.8	0.29
Cardiovascular disease (%)	17.4	22.2	0.63
Stroke (%)	8.3	5.5	0.69
Atrial fibrillation (%)	10.1	5.6	0.54

*BMI, body mass index; MMSE, Mini Metal State Examination*.

*Continuous variables are presented with mean and standard deviation (SD) and categorical variables with proportions. Differences between the groups were tested with the independent sample t-test for continuous variables or chi-squared test for categorical variables*.

### Postoperative Effects on Short-Distance Walking, Exercise Capacity, Strength, and Variables of Activity and Sleep

**Figure 2:**
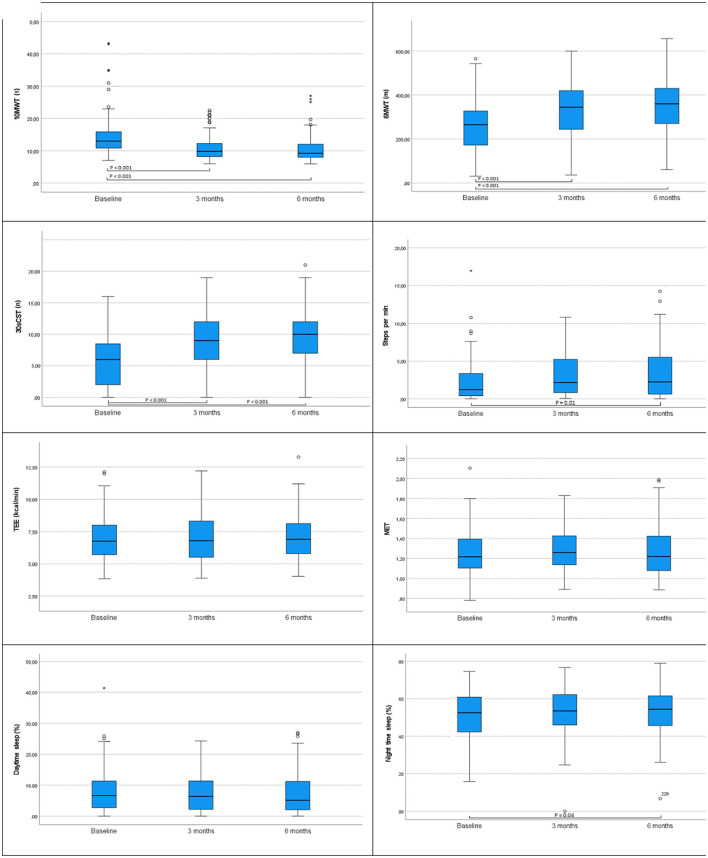
Values at baseline, 3 and 6 months postoperatively for the variables in all patients: 10-m walk test (10MWT), 6-min walk test (6MWT), 30-s chair stand test (30sCST), total energy expenditure (TEE), metabolic equivalent of task (MET), daytime sleep, and nighttime sleep. Significant differences are presented in the figures, *p* ≤ 0.05. Differences were tested with related samples Friedmans two-way analysis of variance (ANOVA) by ranks. Significance values are adjusted by the Bonferroni correction for multiple tests. *N* =109. In the figure of 10MWT, two extreme values are excluded.

The time required to walk 10 m decreased significantly after surgery in a 3-month follow-up (*n* = 94; baseline median 13.0 s, interquartile range (IQR) 10.8–16.1; 3-month median 10.0 s, IQR 8.2–12.3, *p* < 0.001). Also, functional exercise capacity (distance in the 6MWT) increased after 3 months (*n* = 90; baseline median 265.0 m, IQR 171.5–328.5; 3-month median 344.5 m, IQR 243.5–420.0, *p* < 0.001). After 3 months, the functional strength improved as measured by the 30sCST (number of full standings) (*n* = 92; baseline median 6.0, IQR 2.0–8.8; 3-month median 9.0, IQR 6.0–12.0, *p* < 0.001). These results were maintained after 6 months with a trend of further improvement in the 6MWT and 30sCST, but without significant differences to the 3-month assessment (Figure 2). Details are presented in Supplementary Table 1.

The number of steps per minute and the proportion of nighttime sleep (8.00 p.m. to 7.59 a.m.) did not change significantly from baseline to a 3-month follow-up. However, after 6 months, both variables were significantly changed from baseline: steps per minute (*n* = 49; baseline median 1.2, IQR 0.4–3.4; 6-month median 2.4, IQR 0.6–5.7, *p* = 0.01) and the proportion of nighttime sleep (*n* = 55; baseline median 53.0%, IQR 43.0–61.6; 6-month median 53.4%, IQR 45.3–61.4, *p* = 0.04) (Figure 2; Supplementary Table 1).

Total energy expenditure, MET, and the proportion of time spent sleeping during daytime (8.00 a.m. to 7.59 p.m.) did not change significantly from baseline at any of the follow-up assessments (Figure 2, Supplementary Table 1).

None of the variables changed significantly between the 3- and 6-month assessments.

The gait velocity was calculated from the results of the 10MWT and 6MWT. The velocity results of the two tests were highly correlated at baseline (*r* = 0.86), at the 3- (*r* = 0.86), and at the 6-month follow-up (*r* = 0.87) (Table 2).

**Table 2:** Gait velocity calculated from the 10MWT and 6MWT at baseline and 3 and 6 months postoperatively.

	**Gait velocity 10MWT m/s**	**Gait velocity 6MWT m/s**	**Correlation *r***
	***n* =109**	***n =* 109**	
Baseline	0.77 ± 0.26	0.71 ± 0.31 *n =* 106	0.86^*^
3 months postoperatively	1.00 ± 0.27 *n =* 107	0.92 ± 0.33 *n =* 104	0.86^*^
6 months postoperatively	1.05 ± 0.30 *n =* 95	0.95 ± 0.33 *n =* 94	0.87^*^

*10MWT, 10-m walk test; 6MWT, 6-min walk test*.

*Values are presented with mean and SD. Correlations are analyzed with Pearson's correlation*.

* *p < 0.001*.

### Effects of the Physical Exercise Program

There were no significant differences between the exercise group and the control group for any of the efficacy variables in changes from baseline, except for the proportion of daytime sleep. Details are presented in Supplementary Table 1. At a 3-month follow-up, the exercise group decreased more from baseline in the proportion of daytime sleep (*n* = 27; median −1.2%, IQR −4.8–0.2), compared to the control group (*n* = 32; median 0%, IQR 1.3–3.0), *p* = 0.04. At a 6-month follow-up, the differences in changes with respect to baseline disappeared (exercise group: *n* = 25; median −1.0% IQR −4.1–2.3, and the control group: *n* = 37; median 0.3%, IQR −2.4–1.8, *p* = 0.65).

### Correlations Between Postoperative Changes in Short-Distance Walking, Functional Physical Capacity, and Voluntary Walking

After 3 months, the changes with respect to baseline in the 10MWT and 6MWT showed a moderate negative correlation (ρ = −0.49, *p* = 0.01), i.e., the greater the improvement in the 10MWT, the longer the distance in 6MWT after 3 months. This result was maintained with a slightly stronger correlation after 6 months (ρ = −0.54, *p* = 0.01).

Weak negative correlations were seen between changes with respect to baseline in the 10MWT and voluntary walking (steps per minute) after 3 months (ρ = −0.34, *p* = 0.01) with an even weaker correlation after 6 months (ρ = −0.21, *p* = 0.01).

## Discussion

This study reports clear improvements in short-distance walking (10MWT), functional exercise capacity (6MWT), and functional strength (30sCST) 3 months after shunt surgery in iNPH with remaining results after 6 months. In actigraphic recordings, no significant differences from baseline were seen after 3 months, but voluntary walking (steps per minute) improved and the proportion of nighttime sleep increased after 6 months. Changes in the 10MWT and 6MWT were moderately correlated after 3 months, with a slightly stronger correlation at a 6-month follow-up. Correlations between the 10MWT and improvements in voluntary walking were weak at both the 3- and 6-month follow-up assessments.

The gait disturbance in iNPH has been described as broad-based, with short stride length and increased stride variability, and with decreased velocity and low foot to floor clearance (15–17). The gait function improves after shunt surgery (18), and timed short-distance walking tests are used in the evaluation of normal pressure hydrocephalus (NPH) (2, 19–24). However, large studies with a timed 10MWT before and after shunt surgery are scarce (25). The 10MWT performances among the 109 patients in our study were at baseline median 13 s, IQR 11–16, and, at a 3-month follow-up median of 10 s, IQR 8–12. These results partially correspond to a study with 429 patients describing the phenotype of iNPH (20). According to that study, the 10MWT results were slightly higher at baseline (median 15 s, IQR 12–20) and postoperatively (median 12 s, IQR 9–15), but with similar changes compared to our results (20). Gait velocity in the 10MWT at baseline (0.77 ± 0.26 m/s) agrees with the results in a study by Nikado et al. in which fall-related factors were evaluated in 63 patients with iNPH (0.74 ± 0.25 m/s) (21). Normal values in self-selected gait speed (50–79 years) are 1.31 ± 0.20 m/s for women and 1.41 ± 0.21 m/s for men (26). We did not differentiate between men and women, but even 6 months after shunt surgery the walking speed (1.05 ± 0.30 m/s) among patients with iNPH was low compared to healthy individuals (26).

The 10MWT and 6MWT are highly correlated among frail elderly with dementia (*r* = 0.91 *p* < 0.001) (27) and in patients with suspected NPH at baseline (*r* = −0.80, *p* < 0.001) (28). The moderate correlation in our study between changes in the 10MWT and 6MWT may have different causes. Variability in the gait pattern such as decreased velocity, step length and cadence has been reported according to the 6MWT (29). Gait variability may be an important aspect of the gait function in iNPH affecting long-distance walking, but knowledge on variability after 3 and 6 months is lacking. The results from both the 10MWT and 6MWT likely reflect improvements in postural control and gait pattern. However, endurance measured by the 6MWT may not be improved to the same degree after shunt surgery. Heart rate explains most of the variance in the 6MWT, and the motivation to maintain a high intensity during the test will influence the result (30). Heart rate measured in the 6MWT before and 7 days after shunt surgery was unchanged in a small sample of patients with iNPH although velocity increased after surgery (29). Patients with iNPH are used to performing low intensity activities for a long time and may not have the ability to increase intensity during testing in the postoperative period due to persistent poor overall physical condition. The fact that voluntary walking correlated weakly with changes in the 10MWT strengthens this hypothesis.

An interesting finding was the improvement in functional strength regardless of participation in the exercise program after shunt surgery. The 30sCST is intended to evaluate functional lower limb strength displayed when standing upright from a seated position (12). In this movement, the ability to maintain postural control has to be considered. Individuals with iNPH have higher backward–forward trunk sway in quiescent position than healthy individuals, but no significant improvements after shunt surgery have been demonstrated (31–33) although a reduced postoperative presence of retropulsion was reported by Agerskov et al. (20). However, patients with iNPH improve in sway area (34) and voluntary center-of-pressure movements after surgery (33) measured with a force platform. Lower limb strength may improve, but even increased postural control after surgery could have affected the outcome of the 30sCST. Lower limb strength is correlated with gait speed (35), and voluntary center-of-pressure movement is correlated with the gait function (33). The muscle strength of patients with iNPH before and after shunt surgery needs further investigation.

The intensity of daily physical activity does not increase postoperatively, which is confirmed by the nonsignificant changes in TEE and MET. That is, patients do not improve in daily activity levels despite improvements in the clinical assessments. In addition to the prolonged period of physical inactivity, the remaining executive dysfunction and mental behavioral symptoms, including reduced drive and the lack of initiatives (36), may explain these findings. However, an indication of altered behavior in voluntary walking was the improvement after 6 months reported here.

The proportion of nighttime sleep increased significantly after 6 months compared to baseline. This may be interpreted as an improved sleep quality after surgery. However, the differences were small and the result should be interpreted with caution. There are a few studies regarding sleep in iNPH (37, 38). Agerskov et al. showed that the need for sleep decreased after surgery (20). A postoperative improvement in daytime wakefulness has also been reported (39). In our study, there were no changes in the proportion of daytime sleep at any of the postoperative follow-up assessments.

Tailored interventions with professional guidance and ongoing support have been suggested as facilitators to promote physical activity in older adults (40). Meaningful activities and social support, especially from family members, are motivators for maintaining physical activities (40, 41). In our study, the exercise intervention did not affect the measured outcomes. However, the intervention had a high attrition rate, entailing difficulties to detect differences between the exercise group and controls (9). The adoption and maintenance of new behaviors in daily life are complex, and established habits often persist strongly into long-standing behaviors. Habits are automated and trigged by associated cues. Contextual factors have to be considered to underpin behavioral changes and create new habits (42). Such behavioral factors may also explain why improved physical capacities after shunt surgery do not translate directly to improvements in daily physical activity. The lack of postoperative increase, in general, physical activity despite improvements in short-distance walking and physical exercise capacity indicate that patients with iNPH need guidance and motivation regarding meaningful physical activities to utilize the improved functions after surgery. Further research should address how interventions should be tailored to promote physical activity after shunt surgery.

This study has limitations. The outcomes are evaluated before and after surgery, but without a control group of healthy individuals. An additional limitation is that the total group contains a subgroup subjected to a rehabilitation intervention. We had dropouts from the follow-up assessments, and there were internal dropouts from the actigraphic recordings, resulting in small sample sizes.

## Conclusion

Shunt surgery improved short-distance walking, functional exercise capacity, functional strength, and voluntary walking. An exercise program did not affect these outcomes. Short-distance walking was weakly correlated with voluntary walking, indicating that improved physical capacity does not directly translate to increased physical activity. Further research should address how interventions should be tailored to promote physical activity after shunt surgery.

## Data Availability Statement

The raw data supporting the conclusions of this article will be made available by the authors, without undue reservation.

## Ethics Statement

The studies involving human participants were reviewed and approved by Medical Ethical Committee of Linköping, Sweden 2015/250-31. The patients/participants provided their written informed consent to participate in this study.

## Author Contributions

JR, LK, MT, and FL conceptualized and designed the study. MU analyzed the actigraphic recordings, JR, MT, and FL interpreted the results. JR reviewed and edited the manuscript for intellectual content. LK, MU, MT, and FL revised the manuscript and all authors approved the final version for submission. All authors contributed to the article and approved the submitted version.

## Funding

This study was supported by grants from Region Östergötland, the Henry and Ella Ståhls Foundation, the Rune and Ulla Amlöv Foundation, the Edit Jacobson Foundation, the Foundation Hjalmar Svenssons Forskningsfond, the Swedish state under the agreement between the Swedish Government, and the County Councils, the ALF-agreement (#ALFGBG 720121).

## Conflict of Interest

The authors declare that the research was conducted in the absence of any commercial or financial relationships that could be construed as a potential conflict of interest.

## Publisher's Note

All claims expressed in this article are solely those of the authors and do not necessarily represent those of their affiliated organizations, or those of the publisher, the editors and the reviewers. Any product that may be evaluated in this article, or claim that may be made by its manufacturer, is not guaranteed or endorsed by the publisher.
