# Supplementary material for: Physical Capacity and Activity in Patients With Idiopathic Normal Pressure Hydrocephalus
Source: Front Neurol. 2022 Mar 28;13:845976. doi: 10.3389/fneur.2022.845976 (PMC8996117; doi:10.3389/fneur.2022.845976)
Supplement: Supplementary file 1 [file Table_1.DOCX]

Supplementary Material

Table 1│Within group differences and differences between the exercise group and the control group from baseline and the three month and six month follow-up assessments

|  | **Total group**  **__________**  **(n=109)**  **(median; IQR)** | **Within group differences from baseline**  **__________**  ***p*- value** | **Exercise group**  **___________**  **(n=50)**  **(median; min-max)** | **Control group**  **____________**  **(n=59)**  **(median; min-max)** | **Between**  **group differences**  **from**  **baseline**  **__________**  ***p*- value** |
| --- | --- | --- | --- | --- | --- |
| **10MWT (s)**  Baseline  3-month follow-up  Change from baseline  6-month follow-up  Change from baseline  **6MWT (m)**  Baseline  3-month follow-up  Change from baseline  6-month follow-up  Change from baseline  **30sCST (n)**  Baseline  3-month follow-up  Change from baseline  6-month follow-up  Change from baseline  **Steps/min (n)**  Baseline  3-month follow-up  Change from baseline  6-month follow-up  Change from baseline | 13.0(10.8-16.1)  n=109  10.0(8.2-12.3)  n=107  -3.0(-5.0 - -1.1)  n=107  9.23(8.0-12.0)  n=95  -3.0(-5.1 - -1.5)  n=95  265.0(171.5-328.5)  n=106  344.5(243.5-420.0)  n=104  72.5(26.3-106.5)  n=102  360.0(269.3-430.0)  n=94  76.5(40.5-138.0)  n=92  6.0(2.0-8.8)  n=108  9.0(6.0-12.0)  n=108  3.0(1.0-5.0)  n=107  10.0(7.0-12.3)  n=94  3.0(1.5-5.5)  n=93  1.15(0.42-3.37)  n=87  2.23(0.71-5.25)  n=73  0.28(-0.11-1.42)  n=59  2.41(0.63-5.72)  n=73  0.39(-0.18-2.23)  n=62 | <0.001^a^  ^n=94^  <0.001^a^  ^n=94^  <0.001^a^  ^n=90^  <0.001^a^  ^n=90^  <0.001^a^  ^n=92^  <0.001^a^  ^n=92^  0.165^a^  ^n=49^  0.010^a^  ^n=49^ | 12.95(10.8-16.0)  n=50  9.2(8.18-11.8)  n=49  -3.0(-5.33- -1.4)  n=49  9.0(7.6-11.0)  n=43  -3.0(-6.41- - 1)  n=43  271.5(190-330.25)  n=50  360.0(292.0-430.0)  n=47  89.0(40.0-127.0)  n=47  360.00(300.0-438.0)  n=43  90.0(50.0-127.0)  n=43  6.0(3.5-9.5)  n=49  10.0(8.0-12.0)  n=50  3.0(2.0-6.5)  n=49  11.0(7.0-13.0)  n=43  3.5(2.0-6.25)  n=42  1.79(0.52-3.30)  n=37  2.45(1.25-5.22)  n=34  0.57-(0.08-1.78)  n=27  2.61(0.83-5.38)  n=30  0.40(-023-2.31)  n=25 | 13.07(109-16.4)  n=59  10.0(8.2-16.4)  n=58  -2.0(-4.8 - -1.0)  n=58  9.4(8.0-12.8)  n=52  -2.8(-5.0- -1.0)  n=52  253.0(170.0-331.5)  n=56  330.0(240.0-401.5)  n=57  61.0(7.0-96.0)  n=55  350.0(237.0-427.0)  n=51  62.0(26.5-148.5)  n=49  6.0(0-8.0)  n=59  9.0(5.0-11.0)  n=58  2.0(1.0-4.25)  n=58  9.0(5.0-12.0)  n=51  3.0(1.0-5.0)  n=51  1.13(0.38-3.83)  n=50  2.14(0.52-5.45)  n=39  0.09(-0.13-1.18)  n=32  1.87(0.52-6.12)  n=43  0.38(-0.09-2.04)  n=37 | 0.190^b^  0.342^b^  0.092^b^  0.770^b^  0.075^b^  0.156^b^  0.062^b^  0.324^b^ |
| **TEE (kcal/min)**  Baseline  3-month follow-up  Change from baseline  6-month follow-up  Change from baseline | 6.82(5.75-8.05)  n=90  6.79(5.48-8.35)  n=75  0.03(-0.26-0.52)  n=63  6.90(5.63-8.14)  n=75  0.11(-0.43-0.68)  n=64 | ns  ^n=51^  ns  ^n=51^ | 7.10(5.82-8.28)  n=40  7.08(5.53-8.79)  n=34  0.08(-0.29-0.67)  n=29  6.48(5.73-8.13)  n=31  -0.12(-0.80-0.66)  n=26 | 6.61(5.56-7.96)  n=50  6.67(5.42-8.03)  n=41  -0.01(-0.19-0.53)  n=34  6.92(5.62-8.18)  n=44  0.32(-0.20-0.82)  n=38 | 0.811^b^  0.254^b^ |
| **MET (/min)**  Baseline  3-month follow-up  Change from baseline  6-month follow-up  Change from baseline | 1.22(1.11-1.42)  n=90  1.26(1.14-1.43)  n=75  0(-0.06-0.10)  n=63  1.22(1.09-1.43)  n=75  0.02(-0.09-0.13)  n=64 | ns  ^n=51^  ns  ^n=51^ | 1.23(1.11-1.48)  n=40  1.27(1.16-1.43)  n=34  0(-0.07-0.11)  n=29  1.20(1.10-1.40)  n=31  -0.02(-0.14-0.11)  n=26 | 1.22(1.11-1.42)  n=50  1.25(1.09-1.43)  n=41  -0.01(-0.04-0.10)  n=34  1.28(1.04-1.44)  n=44  0.04(-0.03-0.14)  n=38 | 0.900^b^  0.228^b^ |
| **Daytime sleep (%)**  Baseline  3-month follow-up  Change from baseline  6-month follow-up  Change from baseline | 6.76(3.01-11.34)  n=87  6.16(1.66-11.49)  n=73  -0.38(-3.10-1.97)  n=59  5.14(2.09-11.49)  n=73  -0.54(-2.84-1.91)  n=62 | ns  ^n=49^  ns  ^n=49^ | 7.57(3.22-11.96)  n=37  6.34(2.26-11.66)  n=34  -1.22(-4.81-0.17)  n=27  5.40(2.00-17.20)  n=30  -0.98(-4.10-2.25)  n=25 | 6.19(2.24-10.11)  n=50  6.06(1.54-11.78)  n=39  0(1.34-2.96)  n=32  4.68(2.14-10.97)  n=43  0.25(-2.39-1.76)  n=37 | 0.042  0.649^b^ |
| **Nighttime sleep (%)**  Baseline  3-month follow-up  Change from baseline  6-month follow-up  Change from baseline | 52.99(42.97-61.56)  n=90  53.53(46.17-62.38)  n=80  2.15(-2.06-7.34)  n=69  53.42(45.28-61.37)  n=78  2.15(-3.65-4.86)  n=64 | 0.051^a^  ^n=55^  0.040^a^  ^n=55^ | 53.73(41.34-61.27)  n=39  51.69(43.98-62.16)  n=35  2.47(-4.01-6.07)  n=30  51.04(43.59-59.05)  n=35  1.26(-6.95-4.69)  n=27 | 52.54(44.33-62,44)  n=51  51.69(43.98-62.16)  N=45  1.58(-1.66-8.00)  N=42  55.56(48.04-62.28)  n=43  3.37(-0.60-6.03)  n=37 | 0,343^b^  0.229^b^ |

*10MWT, 10 meter walk test; 6MW, six-minute walk test; 30sCST, 30 seconds chair stand test ; TEE, Total Energy Expenditure ; MET, Metabolic Equivalent of Task; a= Related-Samples Friedmans Two-Way Analysis of Variance by Ranks. Significance values adjusted by the Bonferroni correction for multiple tests. b= Mann Whitney U-test. Values are presented as median and IQR. p ≤ 0.05.*
